# Ovary-Sparing Radiation Planning Techniques Can Achieve Ovarian Dose Reduction for Soft Tissue Sarcoma of the Buttock and Thigh

**DOI:** 10.1155/2017/2796925

**Published:** 2017-09-18

**Authors:** Konstantin A. Kovtun, Wee-Pin Yeo, Catherine H. Phillips, Akila Viswanathan, Elizabeth H. Baldini

**Affiliations:** ^1^Harvard Radiation Oncology Program, Brigham and Women's Hospital/Dana Farber Cancer Institute, Boston, MA, USA; ^2^Department of Radiation Oncology, Brigham and Women's Hospital/Dana Farber Cancer Institute, Boston, MA, USA; ^3^Department of Radiology, Brigham and Women's Hospital/Dana Farber Cancer Institute, Boston, MA, USA

## Abstract

**Background and Objectives:**

Attention to ovary dose is important for premenopausal women undergoing radiation therapy (RT) and must not be overlooked when treating extremity sarcoma. We assessed whether ovary-sparing RT plans could decrease ovary dose without compromising target coverage.

**Methods:**

Standard sarcoma target volumes and organs at risk (OAR) were contoured by a sarcoma dedicated radiation oncologist on CT planning scans for 23 women with thigh or buttock sarcoma. IMRT plans (50 Gy) with and without attempted ovary-sparing were created by an expert sarcoma dosimetrist.

**Results:**

All plans met target coverage goals. Compared to standard plans, ovary-sparing plans had lower mean bilateral ovary doses (MBOD) (652 versus 483 cGy, *p* = 0.007) but higher bone doses (mean V50: 8.5% versus 6.9%, *p* = 0.049) and lower conformity indexes (1.12 versus 1.19, *p* = 0.009). Tumors < 8 cm from the pubic symphysis had significant MBOD reduction with ovary-sparing plans (376 cGy versus 619 cGy, *p* = 0.0184). On multivariate analysis, distance to pubic symphysis and proximal medial thigh site were associated with MBOD reduction with ovary-sparing plan.

**Conclusions:**

For preoperative IMRT, ovary-sparing planning significantly reduces ovarian dose in women with sarcoma of the proximal thigh and near the pubic symphysis.

## 1. Introduction

The median age of diagnosis for soft tissue sarcoma (STS) in women ranges from 15 to 65 years based on histologic subtype [1]. Accordingly, many of these women are of child-bearing age and/or premenopausal. Radiation therapy (RT) is a key component of local management for extremity STS [2–4] and wide clinical target volume (CTV) margins are required for optimal local control [5]. For premenopausal women receiving RT who desire fertility or preserved estrogen production, ovarian dose is an important consideration for functional preservation.

Ovaries are not generally discussed as organs at risk (OAR) when planning radiation therapy for thigh and buttock STS. However, studies have shown that increasing ovarian dose is associated with acute ovarian failure, infertility, and premature menopause [6]. It is estimated that 50% oocyte destruction occurs at doses less than 2 Gy [7]. Additionally, effective sterilization doses are reported to decrease with age with 14.3 Gy leading to ovarian failure in 97.5% of patients [8] and 6 Gy leading to an intermediate risk of dysfunction in the average 30-year-old woman. [9]. Given that the ovary is such a radiosensitive organ, careful attention to ovarian dose is imperative when delivering RT to premenopausal women.

We performed an analysis of intensity-modulated RT (IMRT) plans designed with and without intent to spare ovaries for STS of the thigh and buttock to assess whether the ovary-sparing plans could achieve ovary dose reduction without compromising target coverage and to determine if there are clinical predictors of ovarian dose reduction with the new plans.

## 2. Materials and Methods

### 2.1. Patient Data

The study cohort comprised 23 women with STS of the thigh or buttock treated with preoperative RT between September 2010 and February 2016. With Institutional Review Board approval, medical records were reviewed to ascertain patient and tumor characteristics. Women with prior unilateral or bilateral oophorectomy were excluded. All women had CT simulation scans from the pelvis to the knee. An extremity board was used for immobilization, and patients were positioned in a supine position with either a straight leg or slightly frog-legged position. Bolus was not used.

### 2.2. Definition of Target Volumes and Critical Structures

Standard sarcoma gross target volumes (GTV), clinical target volumes (CTV), and planning target volumes (PTV) were contoured by a sarcoma dedicated radiation oncologist on CT planning scans for all patients. T1 postgadolinium MR series were used to contour GTV. GTV to CTV expansions were typically 3.5 cm in the longitudinal directions and 1.5 cm radially on each axial slice with editing for normal tissue interfaces and as per established consensus guidelines [5, 10]. CTV to PTV expansions were 5 mm in all directions. Target volumes were edited from skin surface 3–5 mm. Standard organs at risk (OARs) including bone, bowel, bladder, and rectum were contoured, and the dosimetrist identified an appropriate strip of limb circumference as an OAR during the planning process.

Bilateral ovaries were contoured as separate structures by two radiation oncologists (Konstantin A. Kovtun and Akila Viswanathan) and one radiologist (Catherine H. Phillips) using CT simulation scans as well as pelvic MR scans when available. Final ovary structures were defined by consensus.

### 2.3. Comparative Intensity-Modulated RT (IMRT) Plans

All patients were initially planned without the presence of ovary contours and without regard to ovary dose (standard plan) by an experienced sarcoma planner (Wee-Pin Yeo). All patients were planned with an IMRT technique to a preoperative dose of 50 Gy in 2 Gy fractions. General sarcoma guidelines for beam arrangement were followed including beam selection in attempt to maximize target coverage and spare a longitudinal strip of limb circumference (<20 Gy) and, to the extent possible, avoid beams traveling through the contralateral limb and abdominal and pelvic structures. Coverage criteria were PTV V95 (volume receiving at least 95% of prescription dose) greater than or equal to 95%. OAR constraints included the following: bone (mean dose < 37 Gy, maximum dose < 59 Gy, V40 < 64%), bowel bag (V45 < 195 cc), rectum (V50 < 50%), and anus/vulva/perineum (V30 < 50%)

**Figure 1:**
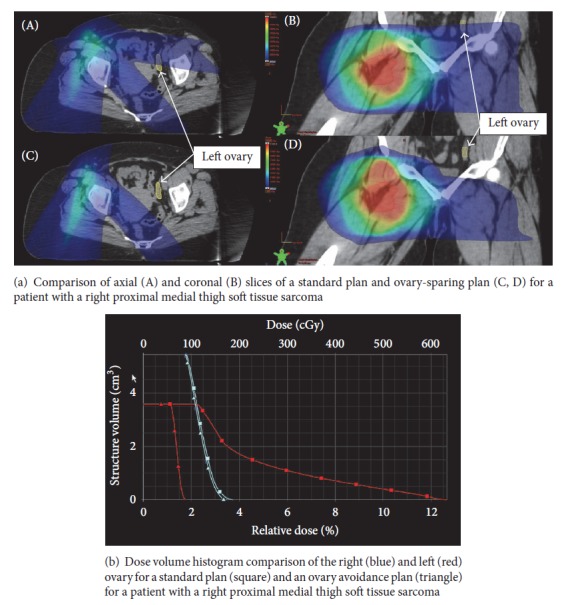


After a 1-month interval during which consensus ovary structures were contoured, the same sarcoma planner produced new plans taking into account the right and left ovary with the goal to keep bilateral ovary dose as low as reasonably achievable (ovary-sparing plan). All other planning criteria were the same for the standard and ovary-sparing plans.  Figure 1 is an example of a standard and ovary-sparing IMRT plan for a patient with a proximal thigh sarcoma showing successful exclusion of the left ovary from the ovary-sparing plan.

### 2.4. Statistical Methods

The distributions of clinical characteristics including age, GTV size, and distance of the proximal-most end of the GTV to pubic symphysis were calculated for the total population and compared according to tumor location (proximal medial thigh, other thigh, and buttock).

Two-sided paired *t*-tests were used for dosimetric comparisons of standard plans and ovary-sparing plans to assess differences in mean bilateral, ipsilateral, and contralateral ovary doses, mean bone dose, bone V50 (volume of bone receiving at least 50 Gy), and conformity index (CI) defined as 100% isodose volume divided by target volume [11]. Dosimetric comparisons by tumor distance from pubic symphysis and anatomic subsite (proximal medial thigh, buttock, or other thigh) for standard and ovary-sparing plans were also assessed by two-sided paired *t*-tests. A cut-off value of 8 cm for tumor distance to pubic symphysis was chosen based on the upper 95% confidence interval of the chance of benefit of an ovary-sparing plan as a function of distance to pubic symphysis.

Univariate and multivariate logistic regression analyses were performed to identify clinical predictors of mean ovarian dose reduction with the use of an ovary-sparing plan. Potential predictors assessed included distance of proximal-most end of GTV to pubic symphysis, largest tumor (GTV) dimension, and anatomic subsite. For univariate analysis, the continuous variables of largest GTV dimension and distance to pubic symphysis were compared between the ovary dose reduction and no reduction groups using a two-sided unpaired *t*-test. For the categorical variable of anatomic subsite, a chi-square *p* value was reported. Multivariate analysis was performed using a logistic regression model with logistic chi-square *p* values reported. Statistical analyses were performed using SAS software, version 9.4 (SAS Institute).

## 3. Results

### 3.1. Ovary Contouring

Planning CT scans included the pelvis for all patients and diagnostic pelvic MR scans were available for 14 of 23 patients (61%). Identification of ovaries on the noncontrast planning CT scans was somewhat difficult, especially as patient age increased. Meanwhile, ovaries were much more easily identified on the MR scans. For patients who did not have a pelvic MR scan available, 4 of 9 (44%) had poor initial ovary contour agreement among the three investigators, whereas for women who had pelvic MR available the rate of poor agreement was 2 of 13 patients (15%).

### 3.2. Comparison of the Distribution of Patient Characteristics Stratified by Subsite

As shown in Table 1, the median age of our study population was 61 years (range: 22–84). There were 13 patients with proximal medial thigh STS, 6 patients with other thigh STS, and 4 patients with buttock STS. Buttock tumors were larger than those of the other sites (median largest dimension of 15.0 cm for buttock versus 7.5 cm for proximal medial thigh versus 7.7 cm for other thigh). Proximal medial thigh tumors were closest to the pubic symphysis (median distance: 5.2 cm versus 16.5 cm for other thigh versus 11.0 cm for buttock).

### 3.3. Dosimetric Comparisons of Standard Plans and Ovary-Sparing Plans

Target volume coverage goals were met for all standard and ovary-sparing plans and OAR constraints for limb circumference, bone, bowel, bladder, and rectum were all met as well. As shown in Table 2, compared with standard plans, the ovary-sparing plans had lower mean bilateral ovary doses (MBOD) (mean dose: 652 cGy versus 483 cGy, *p* = 0.007), lower ipsilateral ovary doses (mean dose: 1072 cGy versus 888 cGy, *p* = 0.03), and lower contralateral ovary doses (mean dose: 232 cGy versus 78 cGy, *p* = 0.075). These lower ovarian doses came at the expense of decreased conformality in the ovary-sparing plans compared with the standard plans (mean conformity index: 1.12 versus 1.19, *p* = 0.009) as well as a small increased bone dose in the ovary-sparing plans (mean V50 bone: 8.5% versus 6.9%, *p* = 0.049).

As shown in Table 3, comparison of ovary-sparing and standard plans by anatomic subsite showed that patients with proximal medial thigh lesions had significant reductions in MBOD with an ovary-sparing plan (MBOD: 293 cGy versus 445 cGy, *p* = 0.008). There were no statistically significant reductions in ovarian dose for other thigh or buttock subsites. Tumors less than 8 cm from the pubic symphysis were also associated with a significant MBOD reduction on ovary-sparing plans (619 cGy versus 376 cGy, *p* = 0.0184). Comparisons of mean contralateral ovary dose (MCOD) based on anatomic subsite and the 8 cm pubic symphysis cut-off showed reductions in MCOD with ovary-sparing, but these comparisons did not reach statistical significance (Table 3).

### 3.4. Univariate and Multivariate Analysis for Predictors of Ovary-Sparing Dosimetric Reduction

Closer distance to pubic symphysis was associated with benefit in MBOD with closer tumors more likely to achieve ovary dose reduction from an ovary-sparing plan. Mean distance to pubic symphysis for plans benefiting from ovary-sparing was 6.3 cm (95% CI: 4.5–8.1 cm) versus 14.1 cm (95% CI: 8.2–19.9 cm) for plans that demonstrated no MBOD reduction (MVA *p* value: 0.0038). Subsite was also associated with ovary dose benefit with 77% of proximal medial thigh and 75% of buttock plans achieving ovary dose reductions (UVA *p* value: 0.054; MVA *p* value: 0.048). Only one of the other thigh subsite plans achieved dose reduction with the ovary-sparing technique. Largest GTV dimension was not associated with a statistically significant ovary dose reduction with the ovary-sparing technique (Table 4).

## 4. Discussion

In this study, we compared standard and ovary-sparing IMRT treatment plans for 23 women with STS of the thigh and buttock. We demonstrated that ovary-sparing treatment plans significantly reduced ovary dose albeit at the expense of decreased plan conformality resulting in a slightly lower conformity index and slightly higher bone V50. Ovarian doses for standard and ovary-sparing plans were highest for tumors located in the buttock, followed by proximal medial thigh and other thigh. The ability to achieve significant ovary dose reduction with ovary-sparing plans compared with standard plans was most evident for proximal medial thigh tumors and tumors located less than 8 cm from the pubic symphysis.

Several points require further discussion. For both women and men interested in fertility and/or who are actively producing sex hormones, the radiation oncologist must be cognizant of minimizing dose to ovaries and testicles, respectively, during the treatment planning process. Since male genitalia are externalized, the importance of attention to testicular placement at the time of simulation, contouring testicles, and setting testicle OAR avoidance constraints tends to be readily apparent. However, for women, since ovaries are in the pelvis, one could imagine that attention to ovaries as an important OAR avoidance structure could be overlooked, particularly when treating tumors of the thigh. This planning study shows that ovarian doses for tumors of the buttock and thigh are frequently high enough to ablate fertility as well as estrogen production, and in several cases, ovarian-sparing plans achieved significant ovary dose reduction. Furthermore, contouring ovaries on noncontrast planning CT scans is often difficult. The availability of pelvic MR scans optimizes the ability to localize ovaries on the simulation scan.

Further study is needed to determine how specific ovary doses might translate into clinical outcomes such as infertility or early menopause. For example, for proximal medial thigh tumors, ovary-sparing plans reduced MBOD from 445 cGy to 293 cGy and reduced MCOD from 167 cGy to 70 cGy. While it is likely that these dose differences would affect ovary function based on data for oocyte radiation sensitivity at 2 Gy [7], further clinical correlation is needed to assess whether such dose differences would be associated with meaningful clinical endpoints such as fertility and/or estrogen production. This is a complicated topic to study. Many factors in addition to radiation dose affect oocyte number and function particularly if systemic therapy is used [12–14]. For example, oocyte number and function decrease with advancing age [15]. Furthermore, since ovaries are paired organs, as is the case for kidneys, potentially acceptable dose constraints will likely vary from maximal sparing of one ovary to a certain dose delivered to both ovaries as series have shown that, in patients receiving ovarian doses of at least 15 Gy excluding at least one ovary, approximately half of the patients developed ovarian dysfunction as opposed to all patients where the contralateral ovary is not spared [16].

There are several limitations of this study. There was heterogeneity of sites and relatively small case numbers. Median age was 61, and thus many of the patients in the sample were postmenopausal. This may partly explain the difficulty encountered with contouring ovaries on some of the planning CT scans. Despite the advanced age of many of the patients in our series, we feel that the ovary-sparing planning exercise was still valid. In addition, the ovarian doses reported are specific to the standard and ovary-sparing plans generated by one experienced dosimetrist. Other planners would likely generate plans with some variations to those reported herein. Lastly, calculation of peripheral dose with treatment planning systems is not always correct and ovary doses so calculated should always be interpreted with caution. However, the point of this report was to highlight the importance of paying attention to ovary dose in premenopausal women receiving radiation in proximity to the ovaries and to show that, with attention to ovary-sparing, for many cases, ovarian dose can be reduced. Despite the above limitations, our study demonstrates the feasibility and potential benefits of ovary avoidance plans in appropriately selected patients.

The findings of our report serve to reinforce the following practice guidelines for any premenopausal woman about to undergo radiation to a site in proximity to the ovaries (trunk, abdomen, pelvis, buttock, and thigh):

1. For women interested in future fertility, a reproductive endocrinology consult is recommended.
2. A diagnostic pelvic MR should be performed to help delineate ovary location on the planning CT scan.
3. The simulation CT scan should include the whole pelvis so ovaries can be contoured and ovary dose calculations performed.
4. Treatment planning beam arrangements should be chosen with ovary-sparing in mind. Target volume coverage should not be compromised. However, it is reasonable to accept minor trade-offs of other OAR constraints in attempt to minimize ovarian dose.
5. For custom prescription templates with OAR constraints, inclusion of ovary and testicle constraints on all templates of trunk, abdomen, pelvis, and extremity is recommended in order to minimize the chance that ovary or testicle dose assessment could be inadvertently overlooked.

## 5. Conclusion

Ovary-sparing planning techniques significantly reduce ovarian dose in women with STS of the proximal thigh or tumors less than 8 cm from the pubic symphysis. Further study is needed to determine accurate ovary constraints, likely stratified by patient age, associated with infertility and premature ovarian failure. In the meantime, when delivering radiation to the trunk, abdomen, pelvis, or thigh for premenopausal women, ovary dose should be calculated and treatment plans that minimize ovary dose selected.

## Figures and Tables

**Table 1:** Patient characteristics (*n* = 23).

Clinical characteristics	Proximal medial thigh (*n* = 13)	Other thigh (*n* = 6)	Buttock (*n* = 4)	All patients (*n* = 23)
Median age (years) (range)	55 (38–76)	58 (22–84)	65 (49–70)	61 (22–84)
Median largest GTV dimension (cm) (range)	7.5 (2.4–15.8)	7.7 (2.5–15)	15.0 (7.9–20.9)	8.3 (2.4–20.9)
Median distance of tumor to pubic symphysis (cm) (range)	5.2 (1.5–10.9)	16.5 (6.9–27)	11.0 (9.3–12.7)	8.0 (1.5–27)
Pelvic MRI available, number (%)	8 (61%)	2 (33%)	4 (100%)	14 (61%)

MRI: Magnetic Resonance Imaging; cm: centimeter.

**Table 2:** Dosimetric comparisons of standard plans and ovary-sparing plans (*n* = 23).

	Standard plan	Ovary-sparing plan	*p* value
Mean bilateral ovary dose (MBOD, cGy) (SD)	652 (100)	483 (84)	0.007
Ipsilateral ovary dose, mean (cGy) (SD)	1072 (1606)	888 (1596)	0.03
Contralateral ovary dose, mean (cGy) (SD)	232 (460)	78 (86)	0.075
Mean bone dose (cGy) (SD)	161 (77)	165 (79)	0.19
Mean bone V50 (SD)	6.86% (7.70)	8.45% (10.1)	0.049
Conformity index, mean (SD)	1.12 (0.07)	1.19 (0.13)	0.009

MBOD: mean bilateral ovary dose; SD: standard deviation; cGy: centigray.

**Table 3:** Dosimetric comparison of mean bilateral ovary dose (MBOD) and mean contralateral ovary dose (MCOD) for standard plans and ovary-sparing plans by anatomic location (*n* = 23).

Tumor distance from pubic symphysis	*N*	Standard plan	Ovary-sparing plan	*p* value
Tumor < 8 cm from pubic symphysis, MBOD (cGy) (SD)	10	619 (765)	376 (540)	0.018
Tumor ≥ 8 cm from pubic symphysis, MBOD (cGy) (SD)	13	678 (1184)	566 (1022)	0.17
Tumor < 8 cm from pubic symphysis, MCOD (cGy) (SD)	10	249 (575)	72 (109)	0.128
Tumor ≥ 8 cm from pubic symphysis, MCOD (cGy) (SD)	13	211 (277)	86 (442)	0.222
*Tumor subsite, MBOD (cGy)*				
Proximal medial thigh (SD)	13	445 (721)	293 (494)	0.008
Other thigh, MBOD (SD)	6	95 (217)	19 (309)	0.33
Buttock, MBOD (SD)	4	2161 (1170)	1800 (1116)	0.20
*Tumor subsite, MCOD (cGy)*				
Proximal medial thigh (SD)	13	167 (255)	70 (50)	0.12
Other thigh, MBOD (SD)	6	8.8 (15.6)	7.2 (11.6)	0.36
Buttock, MBOD (SD)	4	781 (881)	210 (104)	0.26

MBOD: mean bilateral ovary dose; SD: standard deviation; cGy: centigray.

**Table 4:** Univariate and multivariate analysis of likelihood of reduction in mean bilateral ovary dose (MBOD) with an ovary-sparing plan.

	Reduction in MBOD	No reduction in MBOD	UVA *p* value	MVA *p* value
*Mean distance to pubic symphysis (cm) (95% CI)*	6.3 (4.5, 8.1)	14.1 (8.2, 19.9)	0.0079^*∗*^	0.0038^∧^
*Site, number (%)*				
Proximal medial thigh	10 (77%)	3 (23%)		
Buttock	3 (75%)	1 (25%)		
Other thigh	1 (17%)	5 (83%)	0.054^#^	0.048^∧^
*Largest GTV dimension (cc) (95% CI)*	8.7 (5.6, 11.8)	9.5 (6.0, 13.0)	0.72^*∗*^	0.054^∧^

MBOD: mean bilateral ovary dose; SD: standard deviation; UVA: univariate analysis; MVA: multivariate analysis; cm: centimeter; cc: cubic centimeter. ^*∗*^*t*-test *p* value.  ^#^Fisher's exact *p* value. ^∧^Logistic regression chi-square *p* value.
